# Gut Microbiota Status in COVID-19: An Unrecognized Player?

**DOI:** 10.3389/fcimb.2020.576551

**Published:** 2020-11-26

**Authors:** Sabrina Donati Zeppa, Deborah Agostini, Giovanni Piccoli, Vilberto Stocchi, Piero Sestili

**Affiliations:** Department of Biomolecular Sciences, University of Urbino Carlo Bo, Urbino, Italy

**Keywords:** SARS-CoV-2, COVID-19, microbiota-virus interactions, polypharmacy, microbiota manipulating, preventive therapeutic strategies, gut microbiota, gut-lung axis

## Abstract

Infection with the SARS-CoV-2 virus causes cardiopulmonary and vascular complications, ranging in severity. Understanding the pathogenic mechanisms of the novel SARS-CoV2 infection and progression can provide potential novel targets for its prevention and/or treatment. Virus microbiota reciprocal interactions have been studied in a variety of viral infections. For example, the integrity of Coronavirus particles can be disrupted by surfactin, a bacterial surface molecule that targets other viruses, including that of influenza A. In this light, intestinal microbiota likely influences COVID-19 virulence, while from its side SARS-CoV-2 may affect the intestinal microbiome promoting dysbiosis and other deleterious consequences. Hence, the microbiota pre-existing health status and its alterations in the course of SARS-CoV-2 infection, are likely to play an important, still underscored role in determining individual susceptibility and resilience to COVID-19. Indeed, the vast majority of COVID-19 worst clinical conditions and fatalities develop in subjects with specific risk factors such as aging and the presence of one or more comorbidities, which are intriguingly characterized also by unhealthy microbiome status. Moreover, these comorbidities require complex pharmacological regimens known as “polypharmacy” that may further affect microbiota integrity and worsen the resilience to viral infections. This complex situation may represent a further and underestimated risk with regard to COVID-19 clinical burden for the elderly and comorbid people. Here, we discuss the possible biological, physiopathological, and clinical implications of gut microbiota in COVID-19 and the strategies to improve/maintain its healthy status as a simple and adjunctive strategy to reduce COVID-19 virulence and socio-sanitary burden.

## Introduction

SARS-CoV-2 is a novel RNA betacoronavirus (Corman et al., 2020; Zhu et al., 2020), similar to SARS-CoV, first reported in the province of Hubei, in China in November 2019 and rapidly spreading in the World (Heymann and Shindo, 2020). This pandemic disease, called CoronaVIrus Disease 19 (Covid-19) causes different symptoms with extremely variable consequences, from benign to fatal*** *** (Rabi and Al Zoubi, 2020). While many infected individuals remain asymptomatic or show only mild upper airways symptoms, others develop pneumonia and acute respiratory distress syndrome (ARDS) requiring intubation in intensive care unit (ICU) and may undergo complications that can be fatal (Chen et al., 2020). In COVID-19, the occurrence of pneumonia is a critical event discriminating asymptomatic or mild cases, from those with moderate or severe disease. Alongside respiratory problems, including or not pneumonia, infection with SARS-CoV-2 virus causes vascular complications, shock, acute kidney injury, and thromboembolic complications. Several studies based on observation and autopsies revealed an infection of endothelial cells leading to vasoplegia, vascular thrombosis, pulmonary edema, endothelial sloughing, and abnormal regulation of pulmonary perfusion (Piva et al., 2020). Hypoxemia is often refractory to oxygen supplementation and requires invasive mechanical ventilation and intensive care hospitalization for a long time, with a great stress for healthcare systems unable to counteract COVID-19 pandemia. Furthermore, many mechanically ventilated patients develop multi-organ failure syndrome that require prolonged hospitalizations (Quah et al., 2020).

The different responses to the virus attack could be explained by an adaptive immune system that is not efficient enough, or/and pneumonia may start before the immune system responds. The first line defense against SARS-CoV-2 is the innate immunity, whose response is triggered within a few hours of the infection unlike the adaptive response. The natural history of disease is determined by this first interaction between host’s innate immunity and SARS-CoV-2, and by the exposure in the course of the following two weeks, i.e., whether infection will be efficiently blocked in upper airways or it will reach the lungs, and/or other distal organ systems, including gut and its microbiota (Raoult et al., 2020). The development of a defined innate and adaptive immune system, and the maintenance of immune tolerance, is accomplished together with the acquisition of the complex gut microbiota. The gastrointestinal tract hosts a complex, highly diverse microbial ecosystem that interplays with the host, ensuring the establishment and persistence of immune homeostasis (Maslowski and Mackay, 2011; Thaiss et al., 2016; Blander et al., 2017). Humans and their gut microbiota form a composite organism, the so-called holobiont, and the combined genome of all bionts is the hologenome (Postler and Ghosh, 2017). These complex communities of microbes provide an important genomic and enzymatic capability and play a key role in the induction, development and function of the immune system that, in turn, protects from pathogens and sustained tolerance to innocuous antigens, preserving the ecology of microbiota. The important homeostatic relationship between the host immune system and gut microbiota plays a pivotal role to calibrate the threshold of activation of cells and tissues promoting responses to infections (Belkaid and Harrison, 2017).

## Gut-Lung Axis

**Figure 1:**
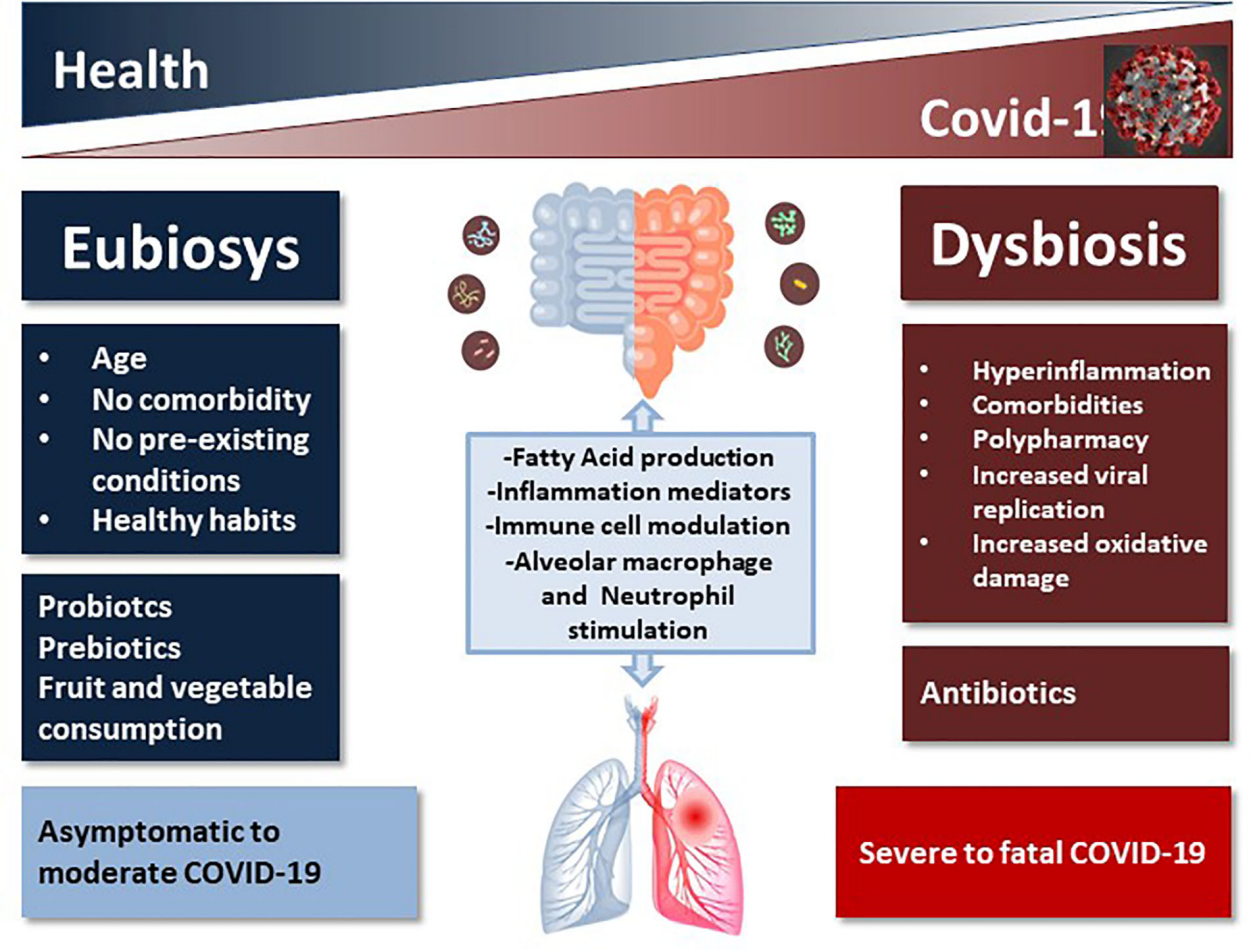
Schematic representation of the impact of healthy vs. unhealthy microbiota on COVID-19 outcome. The complex intercommunication through the gut-lung axis might be important in predetermining the susceptibility of airways to SARS-CoV-2 infection and COVID-19 clinical outcome as a function of the inter-individual variations in microbiota composition and richness.

Dysbiosis and gut microbial metabolites influence immune responses, inflammation, and disease development in the lungs (**Figure 1**). Although still underscored, these conditions might count in large amounts with regard to COVID-19 severity. Gut microorganisms are able to regulate mucosal sites distal from the intestine through its metabolites such as short chain fatty acids (SCFAs) that can reach other organs *via* the bloodstream to exert immune regulation and induction of immunoglobulins, and anti-inflammatory effects (Zhang D. et al., 2020). A healthy microbiota can counteract respiratory tract infection including the influenza A virus (IAV) (Steed and Christophi, 2017; Bradley et al., 2019; Moriyama and Ichinohe, 2019) and *Streptococcus* pneumonia (Schuijt et al., 2016), modulating the functions of effector immune cells, including alveolar macrophages and neutrophils through nucleotide-binding oligomerization domain (NOD)-like receptor agonists (Sencio et al., 2020). Like the enteric system, the respiratory microbiome is complex, with differentiated bacterial communities belonging to *Bacteroidetes*, *Firmicutes*, and *Proteobacteria* phyla inhabiting each niche. Also the respiratory microbiome plays a protective role in immunity (Zhang D. et al., 2020). Influenza can influence pathogenic bacterial adherence to respiratory cells, increasing infection and disease *in vivo* (Schuijt et al., 2016), suggesting an interaction between viral pathogens and bacteria not only in gut, but also in the respiratory tract.

Han et al. (2020) have investigated if the SARS-CoV-2 infection affects lung microflora, which contributes to COVID-19 complications. The results obtained show that the infection of SARS-CoV-2 can deeply change the lung microbiota. Severe microbiota dysbiosis was found in Covid-19 subjects, with a high presence of pathogenic species such as *Klebsiella oxytoca*, Lactic Acid Bacteria, *Faecalibacterium prausnitzii*, and *Tobacco mosaic virus* (TMV).

A dangerous inflammatory environment was found in the lungs, significantly correlated with *Rothia mucilaginosa*, TMV level and SARS-CoV-2. This first evidence suggest an involvement of lung microbiota in the infection mechanism of SARS-CoV-2 and can contribute to the understanding the treatment and complications of this disease (Han et al., 2020).

Among respiratory infections caused by viruses, the upper respiratory tract infections (URTIs) are very common. Intestinal microbiota influences respiratory health, and recent studies demonstrated that gut health-promoting products, such as probiotics and prebiotics, might reduce susceptibility to URTI (Donati Zeppa et al., 2019). The cross-talk between gut microbiota and lungs is referred to as the “gut-lung axis” (Keely et al., 2012) and seems to be bidirectional: gut microbial metabolites can impact the lung through blood and when inflammation occurs in the lung, it can affect the gut microbiota as well (Anand and Mande, 2018; Dumas et al., 2018) (**Figure 1**). Infections, inflammation and metabolic disorders can cause dysbiosis that can modify disease outcomes also in distant organs, such as the respiratory tract, as demonstrated by transfer experiments with dysbiotic microbiota (Sencio et al., 2020), thus creating a vicious circle. Respiratory infections are associated with a change in the composition of the gut microbiota (Groves et al., 2020) and this suggests that even the novel SARS-Cov2 might also have an impact on the gut microorganisms. One of the serious clinical manifestations of Covid-19 is pneumonia and progression to ARDS, in which gut microbiota plays an important role (Dickson, 2018), especially in elderly, immune-compromised patients (Lake, 2020). Angiotensin converting enzyme 2 (ACE2), an enzyme located in the outer surface, mediates SARS-CoV-2 entry in cells and the viral replication (Xu et al., 2020). ACE2 is present in most organs in the arterial and the venous endothelial lining, the arterial smooth muscle cells and the cholangiocytes; its expression is particularly high in renal, cardiovascular, and gastrointestinal tissues, indicating that COVID-19 may involve multiple organs, causing also extrapulmonary symptoms (Hamming et al., 2004). ACE2 is highly expressed in small intestinal enterocytes (Zhang W. et al., 2020), regulates intestinal inflammation, and is involved in diarrhea. COVID-19 appears to primarily spread through respiratory droplets and secretions, but the gastrointestinal tract could be another potential route of infection, since in 10%–20% of cases of COVID-19 (SARS-CoV-2), gastrointestinal disorders are associated with respiratory symptoms (cough, dyspnea). Recently, SARS-CoV-2 has been detected in the stool of COVID-19 patients (Yuen et al., 2020), leading to hypothesize an involvement of gastrointestinal tract in infection. Kopel et al. reported that in approximately 50% of COVID patients, the virus is also found in the feces with negative oral swab, leading to hypothesize not only that there is a replication and therefore an activity in the intestine, but also a greater permanence of the virus (Kopel et al., 2020).

## Microbiota-Virus Interaction

Microbiota-virus interactions have been studied, as well as the positive and negative effects of microbiota on viruses (Robinson and Pfeiffer, 2014). Bacterial surfaces are able to interact with viral proteins, which differ for structures and folds. Viral infection is facilitated by the interaction with the main bacterial envelope components, the lipopolysaccharides (LPS) in Gram-negative and peptidoglycans (PG) in Gram-positive. Poliovirus and other viruses, including *Reovirus*, mouse mammary tumor virus, and murine norovirus, have been shown to use both LPS and PG to enhance their thermostability, receptor affinity and similar mechanisms to facilitate *in vivo* infection (Karst, 2016). Together, these results indicate a key role for commensal bacteria in improving viral adherence, stability and infectivity toward eukaryotic cells. On the other hand, microbiota could confer protection against viral infection by inducing the immune response to avoid infection. Microbiota impact directly and indirectly on virus biology and in turn eukaryotic viruses can influence bacterial biology (Neu and Mainou, 2020). SARS-CoV and Middle East respiratory syndrome coronavirus (MERS-CoV) originated in the bat enteric system (Drexler et al., 2014) maybe using commensal bacteria. CoVs strains could have exploited bacteria during their emergence to promote infection since these commensals are present in good amounts also in the respiratory tract (Domcke et al., 2015). The absence of LPS binding toll-like receptor (TLR) 4 resulted in augmented disease (Totura et al., 2015), due to the TLR pathways role in immunity to SARS-CoV. Johnson et al. (2019) explored the relationship between bacterial surface components and CoV infection, and found that PG from *Bacillus subtilis* reduced CoV infectivity. The molecule responsible for CoV inhibition is surfactin, a cyclic lipopeptide (CLP), that presents dose and temperature dependent virucidal properties. It is known that surfactin can reduce infection by other enveloped viruses, such as that of influenza A, Zika, Dugbe, Nipah, Crimean-Congo hemorrhagic fever, chikungunya, Mayaro, Una, and Ebola viruses (Johnson et al., 2019). This evidence confirms the role for commensal bacteria in increasing or decreasing viral infection, suggesting the importance of microbiota in viral pathogenesis and treatment.

Antimicrobial peptides (AMPs) have been proposed as an alternative therapeutic treatment against MERS-CoV infection by Mustafa et al. (2018). Peptide therapeutics and their mimetics—more than 140 peptides are being evaluated in clinical trials—represent a promising strategy to counteract pathogens potentially including coronavirus and SARS-CoV2 (Mustafa et al., 2018). They can inhibit protein-protein interactions, can be used for diseases difficult to target, and show little side effects and high specificity; furthermore many peptide inhibitors have been demonstrated to counteract efficiently viruses (Melnik et al., 2011). Antiviral mechanisms of peptide action are virolysis, blockage of host cell receptors and of viral fusion or replication, and induction of adaptive immune response. Peptides studied as anti-coronavirus includes mainly peptides inhibiting virus-host cell fusion, that can act on RBD interaction, preventing HR1 and HR2 from forming a fusion-active core or cleaving the S protein (Du et al., 2009) and peptides inhibiting viral entry and replication. Peptides inhibiting assembly and release of virus could be a good target when these processes will be better known. Even if peptides are promising, there are still no treatments or vaccines for COVID-19 approved by the U.S. Food and Drug Administration. Zhou F. et al. (2020) analyzed fecal microbial alterations in 15 patients affected by COVID-19 finding an interesting association with disease severity. There was a correlation between high severity and high baseline abundance of *Coprobacillus*, *Clostridium ramosum*, and *Clostridium hathewayi*, and low *Faecalibacterium prausnitzii* and *Alistipes onderdonkii* levels. The exposure to antibiotics prevented both the enrichment of opportunistic pathogens and positive symbiotic bacteria.

The patients’ fecal samples were tested for viral presence and 11 were positive upon admission, of these, 6 were still positive at the time of hospital discharge. Over time, 14 bacterial species were associated with the fecal viral load, *Bacteroides dorei, Bacteroides thetaiotaomicron*, and *Bacteroides massiliensis*. *Bacteroides ovatus*, known to downregulate expression of ACE2 in murine gut, showed an inverse correlation, while the *Firmicutes Erysipelotrichaceae bacterium2_2_44A a positive correlation*. The results suggest that dysbiosis lasts even in remission (Zuo et al., 2020).

Since the intestinal microbiome is likely to influence COVID-19 severity, and COVID-19 impact the intestinal microbiome with an increase in opportunistic pathogens, the microbiota health might be important to counteract this disease (**Figure 1**).

## Role of Microbiota in COVID-19 Inflammatory Complications

The destruction of lung cells induced by SARS-CoV-2 infection elicits a local immune response involving macrophages and monocytes recruitment, release of cytokines, and prime adaptive T and B cell intervention. This response is usually decisive, but sometimes, it can be dysfunctional leading to severe lung inflammation and cytokine storm. Severe disease patients have high levels of interleukin-2, interleukin-7, and interleukin-10 (IL-2, IL-7, and IL-10), granulocyte colony–stimulating factor (G-CSF), interferon-γ-inducible protein-10 (IP-10), monocyte chemoattractant protein-1 (MCP1), macrophage inflammatory protein 1α (MIP1α) and tumor necrosis factor (TNF) (Huang et al., 2020) together with increased IL-6 levels (Zhou F. et al., 2020). Notably, a higher percentage of CD14+CD16+ inflammatory monocytes, secreting MCP1, IP-10, and MIP1α, was found in peripheral blood of patients with severe disease than patients with mild disease (Zhou Y. et al., 2020). A great variability in the susceptibility of the healthy individuals to the COVID-19 has been observed during this pandemia. Recently, Gou et al. (Gou et al., 2012) obtained important results that could explain the diverse susceptibility among different groups of people suggesting the potential biological mechanism behind these different virus-responses. Twenty blood proteomic biomarkers predicting severe progression of COVID-19 have been identified and this proteomic risk score is positively associated with proinflammatory cytokines mainly among older, but not younger, individuals. Gut microbiota features are found to be highly correlated with proinflammatory cytokines in 366 individuals.

Complications of COVID-19 are more frequent in people with a pro-inflammatory condition and/or an impaired immune response. Low-grade chronic systemic inﬂammation accompanies several comorbidities such as obesity, atherosclerosis, type 2 diabetes, and hypertension that adversely affect the outcomes of patients with COVID-19 (Chiappetta et al., 2020). The gut-microbial–host-immune axis is likely to play a significant role for inflammatory status, and fecal metabolomics analysis by Gou et al. (2020) revealed amino acid-related pathways linking gut microbiota to inflammation and COVID-19 severity. Increased low-grade inflammation is related to lower bacterial diversity. Furthermore, gut microbiota modification during ageing can trigger inflammation; transferring gut microbiota from old mice to young germ-free mice triggers responses mimicking “inflammaging” (Fransen et al., 2017) that includes higher expression of pro-inflammatory cytokine genes, such as TNF-α, and increased circulation of pro-inflammatory bacterial compounds probably linked to permeabilization of the intestinal epithelium by inflammation. Chronic inflammation can cause dysbiosis that, in turn, can cause altered epithelial functioning and consequent disease and infection. LPS are endotoxins derived from the outer cell membrane of Gram-negative bacteria, triggering inflammation-related processes, when endotoxemia is present (Gunness and Gidley, 2010). Dysbiosis may affect gut permeability leading to increases in the systemic levels of bacterial products such as LPS that are able to cross the gastrointestinal mucosa *via* leaky intestinal tight junctions (Gunness and Gidley, 2010). Also indole, one of the major tryptophan-derived microbial metabolites (Russell et al., 2013), produced by the action of bacterial tryptophanase, can interact with inflammation-related processes in the human host (Venkatesh et al., 2014); furthermore, indole-3-propionate indirectly limits LPS infiltration, reducing metabolic endotoxemia and host inflammation (Cani et al., 2012).

Microbiota can metabolize resistant starches and dietary fibers through fermentation, providing SCFAs mainly acetate, propionate, and butyrate, that are used as energy by intestinal epithelium cells and can strengthen intestinal barrier integrity, preventing LPS to induce inflammation (Ohata et al., 2005; Peng et al., 2009; Corrêa-Oliveira et al., 2016). The mechanisms are not completely known, but SCFAs regulates several processes including gene expression, since they possess a histone deacetylase (HDAC) inhibitory activity, chemotaxis, proliferation, differentiation, and apoptosis (Corrêa-Oliveira et al., 2016). Among receptors for SCFAs, free fatty acid receptor 2 (FFAR2) is expressed in intestinal tract epithelial cells and immune system cells, and seems to be involved in cell chemotaxis and activation (Stoddart et al., 2008). It has been demonstrated that high fiber diet, in mice increases blood concentrations of SCFAs attenuating allergic inflammation of the lungs (Trompette et al., 2014). SCFAs may suppress inflammation by reducing migration and proliferation of immune cells, many types of cytokines, and inducing apoptosis, with overall anti-inflammatory effects (**Figure 1**).

## Drugs, Comedications, Polypharmacy, Microbiota, and COVID-19

The vast majority of COVID-19 critical clinical conditions and fatalities occur in elderly people (Lithander et al., 2020). A summa of ageing-related factors concur to render elderly more prone to COVID-19. The presence of one or more comorbidities such as hypertension, hyperlipidemias, cardiovascular diseases, diabetes, and cancer is associated with dramatically increased risk of dying in COVID-19 patients (Lithander et al., 2020). The above comorbidities require chronic and complex pharmacological regimens known as “polypharmacy”, i.e., the use of more than 5 medicines per day. Inevitably, the use of multiple drugs not only implies a proportional increase in drug side effects and adverse reactions, but may also affect microbiome integrity and, consequently, worsen the host capacity of overcoming viral infections (Ticinesi and Milani, 2017), potentially including SARS-CoV-2. Such a situation—***along with other confounding factors and health issues***—may then represent a further and underestimated player with regard to COVID-19 clinical burden, especially for older people.

Unfortunately, only two research articles dated before COVID-19 pandemic dealt with the issue of polypharmacy and microbiota (Ticinesi and Milani, 2017; Vich Vila et al., 2020). Both the studies found that polypharmacy significantly impacts on microbiota composition and that more alterations could be observed at increasing the number of co-administered drugs. Ticinesi and Milani (2017) reported that the microbiota of elderly, comorbid patients was characterized by a general of the relative abundance of pathogenic relevant bacteria such as *Helicobacter*, which possesses numerous extragastric pathological implications, and by the reduction of *Lachnospiraceae* and *Succinivibrionaceae*, which help maintaining host cardiorespiratory health and regulating inflammation, whose exacerbation represents a life threatening complication of COVID-19 (Sestili and Stocchi, 2020; Siddiqi and Mehra, 2020). Ticinesi and Milani (2017) also found a negative correlation between the number of prescribed drugs and the microbiome diversity index; importantly, an increased mortality was observed in the patients with the lowest microbiome diversity index.

### Drug Associations Affecting Microbiota

The interactions between medications and microbiota are not just a number of drug problem: rather, the specific drugs and their combinations should be considered. Unfortunately, apart the cited one by Ticinesi et al., only another study (Vich Vila et al., 2020) focused on the utilization of commonly used drugs on gut microbiota in three independent cohorts from the same geographical area: a general population cohort and two cohorts of inflammatory bowel disease and bowel syndrome patients, respectively. Again, a positive correlation was found between the number of drugs taken and the overall microbiota composition within the three groups. In addition, an analysis between drug- users and non-users was also performed. Single-drug analysis of data highlighted 19 drugs out of 41 which correlated with microbial alterations. With regard to the use of multiple medicines (multi-drug analysis of the data), the presence of laxatives, proton pump inhibitors (PPI), metformin and antibiotics had the highest impact on microbiota composition. On the whole, 47 associations were scored between bacterial relative abundance and the contemporaneous use of 6 drugs. An overall decrease in the genus *Bifidobacterium* was found in multi-drug users, independently of antibiotic assumption. From the functional point of view, 271 bacterial pathways were altered by PPIs, laxatives, antibiotics and metformin.

### Polypharmacy and Resistome

Interestingly, drug assumption affected not only gut community taxonomy and metabolic capacity, but also resistome. Indeed, the number of antibiotic-resistance genes—particularly those responsible for resistance to macrolides—increased across the three cohorts in drug users as compared to participants taking no medication. This effect was promoted not only by antibiotics (see below), but also by 15 non-antibiotic drugs, particularly PPIs and metformin (Zheng et al., 2019). Notably, such an event could thwart the efficacy of azithromycin, one of the most prescribed antibiotics to manage/prevent COVID-19–associated airways superinfections (Ohe et al., 2020).

To explain the mechanisms whereby non-antibiotic drugs induce these alterations, it has been proposed that when a wide variety of drugs are given to the same patient, the taxa capable of metabolizing xenobiotics can benefit from a competitive advantage over the non-metabolizing ones, leading to (dys)microbic significant modifications (Vich Vila et al., 2020). This may have negative consequences on the COVID-19 elderly patients’ immunological resilience and capacity to overcome SARS-CoV-2 infection.

As to antibiotics, more than any other drug class, they promote not only severe gut dysbiosis, but also increase in bacterial resistance through mechanisms involving, among many, gut bacteria communities (Becattini et al., 2016). Hence, a history of antibiotic (mis)use and polypharmacy may promote the selection of resistant commensal strains which constitute a reservoir of transmittable resistance factors in elderly, comorbid population; these factors might be acquired by the pathogens superinfecting COVID-19 patients rendering their eradication more difficult. Unfortunately, the occurrence and the relevance of this phenomenon cannot be quantified yet, and specific retrospective studies should be planned.

### Drugs Used to Treat COVID-19 and Microbiota

The drugs commonly prescribed to treat mild COVID-19 need to be combined to preexisting polypharmacy and may theoretically complicate the range of interactions with microbiota. For instance, NSAIDs may affect microbiota composition, whose alteration seems in turn to affect their gastrotoxicity (Castellsague et al., 2012). Paracetamol—recommended as a safer alternative to NSAIDs—deserves a special mention since it has been widely used in COVID-19. Paracetamol does not alter microbiota composition, but its absorption and bioavailability is greatly increased in dysbiotic patients (Mukhtar et al., 2019), which may then be more prone to its hepatic toxicity and glutathione depletion, two conditions potentially exacerbating the course of COVID-19 (Sestili and Fimognari, 2020).

As discussed above, the prescription of antibiotics to prevent/treat superinfections may further and profoundly affect COVID-19 patients’ microbiota, especially broad spectrum agents. A special mention should however be made of azithromycin—a widely prescribed antibiotic for COVID-19 treatment—since it causes a very rapid reduction in bacterial richness (23%) and Shannon diversity (13%), with microbiota composition shifted primarily in the *Actinobacteria* phylum alongside reduction of abundance in the genus *Bifidobacterium*. Hence, azythromycin—more than other agents—has the potential to rapidly worsen the already weak microbiota status of elderly, comorbid COVID-19 patients.

Glucocorticoids, especially desametasone, are prescribed in COVID-19 as the most effective drugs to contain/reverse hyperinflammation; they are also used by patients to treat pregress underlying diseases. To this regard, two recent studies reported that glucocorticoids induce gut microbial alterations in mice. (Schepper et al., 2019) and humans (Qiu et al., 2019). In human subjects diagnosed for acute transverse myelitis, 3 months treatment with prednisone promoted enrichment in *Firmicutes* and depletion in *Bacteroidetes* (Qiu et al., 2019).

As to antiviral drugs, remdesivir does not affect microbiota while hydroxychloroquine—although it is no more recommended against SARS-CoV-2—in conjunction with doxycycline has been reported to alter microbial community composition (Angelakis et al., 2014).

Overall, polypharmacy-induced alterations of gut bacterial community may represent a further and yet disregarded factor contributing to worsen the already bad microbiome conditions in multimorbid patients (**Figure 1**).

## COVID-19: Therapeutic Approaches for Microbiota Health

Italy is among the first countries in Europe for antibiotic consumption; Spain and France are also very high and last year they consumed even more antibiotics than Italy. Antibiotics are known to cause intestinal dysbiosis and it is therefore plausible that the abuse of antibiotics in these three countries (Italy, Spain, and France), which happens to be among the most affected by the Covid-19 emergency, is a risk factor. Many data will have to be cross-referenced before we can say that antibiotic abuse is somehow also responsible for this tragedy. The comorbidities present among the positive deaths at SARS-CoV-2, such as diabetes, hypertension, atrial fibrillation, dementia, stroke, lead us to ask if it is the disease itself that is a risk factor for Covid-19, or/and the drugs taken to keep them at bay. As reported above, several studies now show that many drugs (metformin, statins, PPI, psychiatric drugs) alter the gut microbiota and these alterations can increase the risk of viral infections. The average age of those suffering from severe Covid-19 complications is quite high, and it’s known that the gut microbiota of the elderly is “fragile”, with an impoverished biodiversity. Furthermore, vitamin D deficiency is almost always observed in the elderly (Agostini et al., 2018) and recently a correlation between COVID-19 progression and vitamin D, as well as a relation between this vitamin and gut microbiota homeostasis, have been observed (Sun, 2018; Rhodes and Subramanian, 2020).

Considering what we have discussed so far, specific supplement recommendations can be suggested to prevent COVID-19 infection or at least decrease the severity of disease. Novel therapeutic strategies that target manipulation of gut microbiome could include probiotics, prebiotics, natural products or diets. Probiotics are live non-pathogenic microorganisms which, when administered in adequate amounts, confer microbial balance, particularly in the gastrointestinal tract (Williams, 2010). Probiotics have been demonstrated to lower the frequency and duration of diarrhea and to regulate humoral and cellular immunity inhibiting the TLR expression and the corresponding signaling pathways (Mishra et al., 2015). TLR, a pattern recognition receptor, is a protein of the natural immune system. TLRs are widely expressed on cell membranes in cells that are involved in the innate and the specific immune system, indicating their importance as part of the defense mechanism of the body. TLRs first recognize the pathogen and trigger a series of molecular cascades leading to an aggravated inflammatory reaction with an abnormal release of cytokines and an increase of mucosal damage (Yao et al., 2017). Furthermore, they show significant antioxidant activity both *in vivo* and *in vitro* (Lin and Yen, 1999; Dowarah and Verma, 2018; Noureen et al., 2019). Luoto et al. demonstrated that the incidence of viral respiratory tract infections, especially those caused by Rhinovirus in preterm infants was significantly lower after early in life assumption of probiotics and prebiotics (Luoto et al., 2014). A reduction of the duration of acute respiratory infections because of probiotics supplementation (specifically *Lactobacillus* and *Bifidobacterium* genera), in otherwise healthy children and adults has been reported by King et al. (2014). *Lactobacillus* and *Bifidobacterium* genera have the strongest antiviral activity against respiratory viruses, in particular against influenza virus type A. This antiviral activity depends on the strain’s specificity and the situation of the host immune system even if the main mechanism of such probiotics is immunomodulation (Al Kassaa, 2017). Similarly, probiotics and prebiotics administration in the early stages of life of preterm newborns has also led to a significant reduction in the incidence of viral infections of the respiratory tract, in particular those caused by Rhinovirus (Luoto et al., 2014). Covid-19 patients often require invasive mechanical ventilation. Interestingly, some authors demonstrated that probiotics administration decreases the need for this procedure in critically ill patients (Zeng et al., 2016). In addition, a pilot study carried out by Morrow et al. (2010) the administration of *Lactobacillus rhamnosus* GG, prevented ventilator-associated pneumonia in a selected, high-risk ICU population. Other probiotics strains, *Bacillus subtilis* and *Enterococcus faecalis*, have also been shown to decrease the need for mechanical ventilation in most serious patients (Zeng et al., 2016) A decrease of specific gut commensal bacteria, *Lactobacillus* and *Bifidobacterium*, has been observed in some Covid-19 patients showing intestinal dysbiosis. These bacteria are known to exert host-beneficial effects such as protection from intestinal infections, stimulation of intestinal function, improved immune response and prevention of excessive growth of *Candida, Pseudomonas, Staphylococcus*, and Escherichia coli during antibiotic treatment (Xu et al., 2020). Kiousi et al. (2019) demonstrated that probiotics can reduce cytokine production either locally or in organs other than intestine, and that their administration can positively influence immune system conditions and respiratory tract infections; in particular prebiotics and probiotics stimulate plasmacytoid dendritic cells (pDCs) *via* TLR9 (Toll-like receptor 9), and, in turn, interferon (IFN) production reduces viral replication and infectivity  (Kiousi et al., 2019). The probiotic *L. lactis* JCM 5805 could also be a tool to enhance anti-viral immunity in humans because it is able to activate pDCs, inducing IFN production *in vitro* (Sugimura et al., 2013). Starosila et al. (2017) isolated from the probiotic strain *Bacillus subtilis* the peptide P18, that causes a complete inhibition of influenza virus *in vitro* and an important antiviral effect in mice. Lung microbiota also contributes to immunological homeostasis and its dysbiosis increases the susceptibility to viral infection can be higher if it is affected by dysbiosis. Furthermore, lung microbiota dysbiosis may promote the development of secondary bacterial infections, increasing the morbidity and mortality in Covid-19 patients (Di Pierro, 2020). A predominant commensal in healthy oral and lung microbiotas is *Streptococcus salivarius* which has been proposed as a possible probiotic (*Streptococcus salivarius K12*) approach to improve oral and lung microbiotas and raise defenses against SARS-CoV-2 by Di Pierro (2020). Fiber and resistant starch are digested by intestinal microorganisms (De Filippis et al., 2016) furnishing energy to the host and improving intestinal health. Inuline, polydextrane, maize fiber, and other prebiotics are known to increase gut diversity and immunity in humans, especially during ageing (Kleessen et al., 1997; Bouhnik et al., 2007). For example, a reduction of IL-6 and insulin resistance has been noted consuming non-digestible carbohydrates from whole grains, while IL10 increased after assumption of butyrylated high amylose maize starch (Keim and Martin, 2014). These beneficial effects are likely to be due to an increased production of SCFAs and a strengthening of the gastro-intestinal associated lymphoid tissue (Schley and Field, 2002). The increase of fiber in diet can also improve the lung microbiota, influencing lung immunity (Trompette et al., 2014). Hence, the use of prebiotics and probiotics should be exploited as a novel and simple strategy to help counteracting viral infections.

Alongside prebiotics and probiotics, other food components display indirect beneficial effects mediated by gut microbiota. Whey and pea protein extracts have been reported to increase *Bifidobacterium* and *Lactobacillus*, and whey is able to decrease the pathogenic bacteria *Bacteroides fragilis* and *Clostridium perfringens* (Świątecka et al., 2011). In addition, fats can influence gut microbiota status: a low-fat diet can increase *Bifidobacterium*, while a high saturated fat diet augments the relative proportion of *Faecalibacterium prausnitzii* (Singh et al., 2017).

Anti-inflammatory drugs are known to contrast Covid-19, and an increase in anti-inflammatory food intake (i.e., vegetables, fruit and fish), together with a reduction in pro-inflammatory foods, such as red meat, processed foods and alcohol, could reduce baseline gut inflammation and be very helpful in managing pandemia (Singh et al., 2017; Zabetakis et al., 2020)

A plant based rich fiber diet is a good source of microbiota accessible carbohydrates which improves intestinal health by producing short fatty acids (SCFA) and provides various health benefits to the host including enhanced immunity (Rishi et al., 2020).

Furthermore, preclinical and clinical data suggest that dietary polyphenols, that are heterogeneous compounds of natural origin present in food items such as vegetables, fruits, cereals, tea, coffee, dark chocolate, cocoa powder, and wine present prebiotic properties and exert antimicrobial activities against pathogenic gut microbes, having benefits in distinct disorders, affecting gut metabolism and immunity and exert anti-inflammatory properties (Kumar Singh et al., 2019).

It is therefore evident that a correct personalized diet can help in improving recovery and clinical outcomes of patients affected with Covid-19.

## Conclusions

Gut microbiota can influence immune response, thereby affecting the disease progression. Both overactive and underactive immune response possibly associated with the gut microbiota status can lead to serious clinical complications in COVID-19. The unhealthy status of microbiota might then represent a still underscored risk factor. Since microbiota can be supported through the assumption of adequate, safe, and inexpensive prebiotics and probiotics, their prescription should be considered as either an adjunctive treatment to limit COVID-19 progression in infected patients, or a preventive strategy for non-infected people at risk in the course of COVID-19 expansion or secondary waves.

## Author Contributions

Conceptualization and original draft preparation: SDZ. Resources and writing: SDZ, DA, and PS. Editing and figure preparation: GP. Supervision: PS and VS. All authors contributed to the article and approved the submitted version.

## Conflict of Interest

The authors declare that the research was conducted in the absence of any commercial or financial relationships that could be construed as a potential conflict of interest.
